# A novel and safe SmartCap^®^ SC101 to develop the COVID-19 mRNA vaccine STP2104 inducing potent immune responses in humans

**DOI:** 10.3389/fimmu.2025.1571092

**Published:** 2025-06-16

**Authors:** Rachel Kim, Tae-Gi Uhm, Jisu Kim, Dayeon Woo, Uk-Il Kim, Xue Meng, Byounggu Yang, Suhyeon Kim, Heejene Kim, Jonghyeon Kim, Sunkyung Yoon, Joo-Young Lee, Byungkyun Kim, Dongheon Cho, Duckho Chang, Young-Hwan Cho, Kanghyun Choi, WonSeok Gwak, Hoon-Woo Lee, Jieun Bang, Elizabeth Hellström, Byoungguk Kim, Kyungjin Kim, Joo-Sung Yang

**Affiliations:** ^1^ R&D Center, ST Pharm Co., Ltd., Seoul, Republic of Korea; ^2^ Division of Clinical Research for vaccine, Center for Vaccine Research, National Institute of Infectious Diseases, Korea National Institute of Health, Korea Disease Control and Prevention Agency, Cheongju, Republic of Korea; ^3^ Be Part Research (PTY) Ltd., Paarl, South Africa

**Keywords:** mRNA vaccine, COVID-19, SARS-CoV-2, STP2104, human clinical trial study, Capping Library Screening, 5’-cap analogue, SmartCap^®^

## Abstract

We have developed a 5′-capping library screening (CLS) method using over 30 different novel cap analogues. The optimal 5′-cap for the coronavirus disease 2019 (COVID-19) mRNA vaccine STP2104 was selected and applied. This is the first report to describe the proven safety of the novel cap analogue, SmartCap^®^ SC101, in humans and emphasize the importance of cap selection. STP2104 demonstrates safety, tolerability, and strong immune responses in humans. After confirming its safety through a GLP toxicity study, STP2104 was administered intramuscularly as a two-dose vaccine, separated by 28 days, in COVID-19-naive, healthy adult volunteers. In this multicenter, open-label, dose-escalation, phase I study with 30 participants (18 to 55 years of age), 15 individuals each were assigned to the low-dose (25 μg) and high-dose (50 μg) cohorts. The primary endpoints were the safety and immunogenicity in all cohorts. During the reporting period of the trial, no serious adverse events were reported. A plaque reduction neutralization test demonstrated an at least 21-fold increase in NAb titers from both cohorts when comparing pre-vaccination to 4-week post-second vaccination. These safety and NAb titer interim results support the efficiency and safety of SC101 and the STP2104 mRNA vaccine, including how STP2104 effectively induces NAb titers against SARS-CoV-2.

## 1 Introduction

Coronavirus disease 2019 (COVID-19) is caused by severe acute respiratory syndrome coronavirus 2 (SARS-CoV-2), which first emerged in Wuhan, China, in late 2019 (1). The novel zoonotic coronavirus was first identified in patients with pneumonia of unknown cause and rapidly spread throughout the world (2). As part of the mechanism of entry of SARS-CoV-2 into host cells, the spike glycoprotein (S) binds to its receptor, human angiotensin-converting enzyme 2 (hACE2), to initiate infections with respiratory and systemic manifestations (3).

Shortly after the emergence of SARS-CoV-2, the World Health Organization (WHO) declared COVID-19 a pandemic in March of 2020, and in 2022, COVID-19 transitioned to the endemic phase (4). To counter the global spread of COVID-19, there was an urgent need for safe and effective vaccines, and among the various types of vaccines that have been developed or are in the process of development (e.g., recombinant protein vaccines, adenoviral vector-based vaccines, DNA vaccines, and mRNA vaccines), mRNA vaccines have demonstrated remarkably effective protection against SARS-CoV-2 (5–7). The efficacy of mRNA vaccines is evidenced by the reduction in incidence rates of both positive SARS-CoV-2 PCR testing and COVID-19-associated hospitalizations such as intensive care unit (ICU) admission without serious safety concerns compared to other types of vaccines (8). During the early stages of the pandemic, the first two vaccines against COVID-19 to receive emergency use authorization (EUA) were nucleoside-modified mRNA vaccines manufactured by Pfizer-BioNTech (BNT162b2) and Moderna (mRNA-1273) (9, 10). The nucleoside-modified COVID-19 mRNA vaccines COMIRNATY^®^ (BNT162b2), SPIKEVAX^®^ (mRNA-1273), and DAICHIRONA^®^ (DS-5670) have already been approved for commercialization, among others, and many are in the process of clinical trials (10–14).

As several new variants of SARS-CoV-2 have been identified, variants with enhanced transmissibility, antigenicity, or immune evasion obtained through the evolution of the virus have continuously caused an increase in immune-breakthrough cases (15). Furthermore, financial burden, supply shortages, and political disputes have caused an unbalanced vaccine distribution globally (16, 17). In order to address the insufficiency in meeting medical needs, it has been crucial to develop an affordable and competitive vaccine candidate using the most up-to-date technology and versatile mRNA platforms (18).

The structural elements of most COVID-19 mRNA vaccines include the 5′-cap, 5′ untranslated regions (UTRs) on both ends, open reading frame (ORF)-encoding proteins [nucleoside-modified coding sequence of the spike protein, including the receptor-binding domain (RBD)], a 3′ UTR, and a poly-A tail, all encapsulated by lipid nanoparticles (LNPs) (19). We have invented novel structures of 5′-capping analogues based on our previous monomer library synthesis experiences and established a capping library consisting of over 30 different cap analogues. There are two different types of capping methods: co-transcriptional capping, commonly used in *in-vitro* transcription (IVT) with the use of cap analogues; and post-transcriptional capping via enzyme RNA 5′-triphosphatase (RTPase). The function of 5′-capping analogues is essential for promoting the stability and transportation of mRNA and protecting it from degradation (19). For these reasons, screening for optimal cap analogues was conducted using the CLS method with three different open reading frames [enhanced green fluorescent protein (eGFP), human erythropoietin (hEPO), and firefly luciferase (fLUC)] in two different cell types in order to select an optimum 5′-capping analogue representing higher protein expression for prophylactic vaccine development. From the library screening, SmartCap^®^ SC101, used for the co-transcriptional capping of mRNA for three reporter proteins, showed consistently higher protein expressions of eGFP, hEPO, and fLUC in both HEK293 T and Huh7 cell lines. This is comparable to the protein expression of mRNA synthesized using a TriLink CleanCap^®^ Reagent AG under ST Pharm’s optimized reaction conditions. In a preclinical *in-vivo* animal study, we applied the SmartCap^®^ SC101 for the development of the STP2104 COVID-19 mRNA vaccine and conducted immunogenicity and challenge experiments. STP2104 encodes the codon-optimized full-length S-type (ancestral) spike protein with exogenous signal peptide, thus improving protein expression and secretion, as well as modified nucleoside N1-methylpseudouridine (m1Ψ) 5′ triphosphate, which is encapsulated with an LNP for efficient delivery, as previously reported (20, 21). The STP2104 prophylactic mRNA vaccine candidate induced potent humoral immune responses, including stronger neutralizing activity and cellular immune responses and good protection efficacy. These results indicate that STP2104 has the potential to be used as an effective LNP-mRNA vaccine against SARS-CoV-2 infection (22). The SmartCap^®^ library can be considered a powerful tool for the selection of 5′-cap platforms for mRNA medicine development and may support the development of future mRNA vaccines.

Prior to the phase I clinical trial, we conducted a repeated-dose GLP toxicity study to evaluate the systemic toxicity of STP2104 when administered intramuscularly to Sprague–Dawley (SD) rats three times in 17 days. After 3 weeks of a treatment-free recovery period, the reversibility or progression of any treatment-related changes or delayed toxicity was assessed. Based on the GLP toxicity results, we designed the clinical study as a two-dose basic vaccine. The multicenter, open-label, dose-escalation, phase I study was conducted among 30 COVID-19-naive, healthy adults (18 to 55 years of age). Based on order of enrollment, 15 participants were assigned to the low-dose-level cohort of 25 μg, while 15 participants were assigned to the higher-dose-level cohort of 50 μg (following dose escalation), with each respective cohort receiving two doses 28 days apart. The primary endpoints were to evaluate the safety and immunogenicity in all dose groups. The interim results of the human clinical study demonstrated highly potent humoral and cellular immune responses; furthermore, no serious adverse events (SAEs) were reported during the study period.

The main focus of our report is to present the human safety and efficacy data for the first human use of the SmartCap^®^ SC101 within the context of our own COVID-19 mRNA vaccine platform. The development of the STP2104 COVID-19 mRNA vaccine was initially planned during the pandemic. Our goal was to expedite the development of this platform using the ancestral strain, with the flexibility to replace it with the spike sequence of the prevalent variant as needed, in response to the timely emergence of new variants.

In this study, we confirmed that SmartCap^®^ SC101-embedded STP2104 provides safe, less toxic, and highly immunogenic protection against SARS-CoV-2 virus infection and minimizes disease severity in humans. Details on human reactogenicity, safety, and tolerability data will be reported sequentially.

## 2 Materials and methods

### 2.1 STP2104 COVID-19 mRNA-LNP vaccine manufacturing process

The SmartCap^®^ SC101 chemical structure, synthesis, and STP2104 manufacturing process were described in another paper (22).

### 2.2 LNP formulation

STP2104 was manufactured by mixing an mRNA solution and lipid solution according to the procedure reported in previously published papers (21, 23–25). Briefly, STP2104 is a COVID-19 mRNA-LNP vaccine that has SAR-CoV-2 spike protein mRNA encapsulated within the LNP. The LNP consists of ionizable lipids [(6Z,16Z)-12-((Z)-dec-4-en-1-yl)docosa-6, 16-dien-11-yl 5-(dimethylamino) pentanoate (Lipid 10) (ST Pharm, Republic of Korea), 1,2-distearoyl-sn-glycero-3-phosphocholine (DSPC, Avanti Polar Lipid, USA), cholesterol (Merck, Germany), and PEG_2000_-c-DMA (ST Pharm)] in a molar ratio of 50:10:38.5:1.5. The lipid components dissolved in ethanol and the mRNA dissolved in acetate buffer (pH 5) were mixed at a 1:1 volume ratio using a T-mixer. The obtained LNPs were diafiltered with PBS and ultrafiltered with a Tris-based sucrose buffer using a tangential flow filtration (TFF) system. Finally, the STP2104 bulk drug substance (DS) was sterile-filtered with a 0.2-μm pore size membrane and fill-finished for drug product (DP) production.

### 2.3 GLP repeated-dose toxicity study

Male and female Sprague–Dawley rats, aged 6–8 weeks, were acclimatized for 1 week before the start of the experiment. The STP2104 vaccine materials were administered via the intramuscular route at a fixed dose of 25, 50, or 100 μg/animal in SD rats three times in 17 days, and the reversibility and any delayed effects following a 21-day recovery period were assessed (**Table 1**).

**Table 1:** Preclinical repeated-dose toxicity study: treatment groups and doses.

Group	1	2	3	4	5
**Compound**	Vehicle (PBS)	STP2104 vaccine	STP2104 vaccine	STP2104 vaccine	LNP alone
**Dose (µg/animal)**	0	25	50	100	0

The dose refers to the amount of mRNA, and LNP is a lipid nanoparticle that serves as a carrier for the mRNA vaccine.

The STP2104 vaccine and LNP were kept at room temperature (15°C to 25°C) for at least 30 min before being injected, and once they were removed from the refrigerator, administration was completed within 2 h. Clinical condition, food consumption, and body weight were monitored during the study. Additionally, ophthalmoscopy, hematology and coagulation (peripheral blood), blood chemistry, urinalysis, macropathology, organ weight, and histopathology investigations were conducted^1^. Hematology parameters (e.g., red and white blood cell counts, hemoglobin, hematocrit, platelet count) were also assessed. The clinical chemistry analysis included liver enzymes (ALT, AST, ALP), kidney function tests (creatinine, urea), and other relevant biomarkers (e.g., bilirubin, albumin, glucose)^1^.

### 2.4 Phase I clinical trial

In phase I of the STP2104–101 clinical study, healthy adults (18 to 55 years of age) were enrolled at sites opened in South Korea and South Africa. The study protocol was approved by the respective Institutional Review Boards (IRBs) and the government and national regulatory authorities of each site’s country. All study procedures were conducted in accordance with the Declaration of Helsinki and were performed following Good Clinical Practices and according to the International Council for Harmonisation of Technical Requirements for Pharmaceuticals for Human Use.

### 2.5 Participants

Adult male and female participants (18 to 55 years of age) in good health with no significant medical history were selected, and no clinically significant abnormalities were found upon physical examination. The main criteria for inclusion included body mass index (BMI) ≥18.0 and ≤30.0 kg/m^2^ at screening, availability during the duration of the study, agreement to avoid blood donation or transfusion during the clinical trial, to abstain from alcohol intake for 48 h both before and after each vaccination, and to use highly effective and medically accepted contraception. The main exclusion criteria included a previous diagnosis of COVID-19 or positive antibodies [immunoglobulin M (IgM) and/or immunoglobulin G (IgG)] to COVID-19 identified at screening (using an Elecsys Anti-SARS-CoV-2 S kit, Roche Diagnostics, Indianapolis, IN, USA); close contact with a person infected with COVID-19 within 14 days before the first vaccination; healthcare workers who directly participated in the care of patients diagnosed with COVID-19 or those at high risk of exposure to SARS-CoV-2; previous vaccination against COVID-19 or participation in an LNP-related clinical trial; history of SARS-CoV or MERS-CoV infection and/or vaccination against them; history of severe allergic reaction, hypersensitivity or allergy, or SAE involving vaccine components; chronic use of steroids within 6 months prior to the first vaccine dose; and the administration of immunosuppressants or other immune-modifying drugs within 6 months prior to the first vaccine dose. In addition, among the exclusion criteria were vaccines administered or scheduled in the period from 4 weeks prior to the first scheduled vaccination to 4 weeks post-second vaccination; suspected or known history of drug abuse or alcohol abuse within 6 months prior to the first vaccination; the administration of other investigational drugs or clinical investigational devices within 6 months prior to the first vaccination; receiving immunoglobulin or blood-derived products within 3 months before the first vaccination or planned during the course of the study; pregnant or lactating women; and several other criteria. Prior to the study, women of childbearing potential were required to have a negative pregnancy test to participate in the study, and both male and female participants were required to use approved highly effective methods of contraception, as stated in the study protocol.

### 2.6 Study design

**Figure 1:**
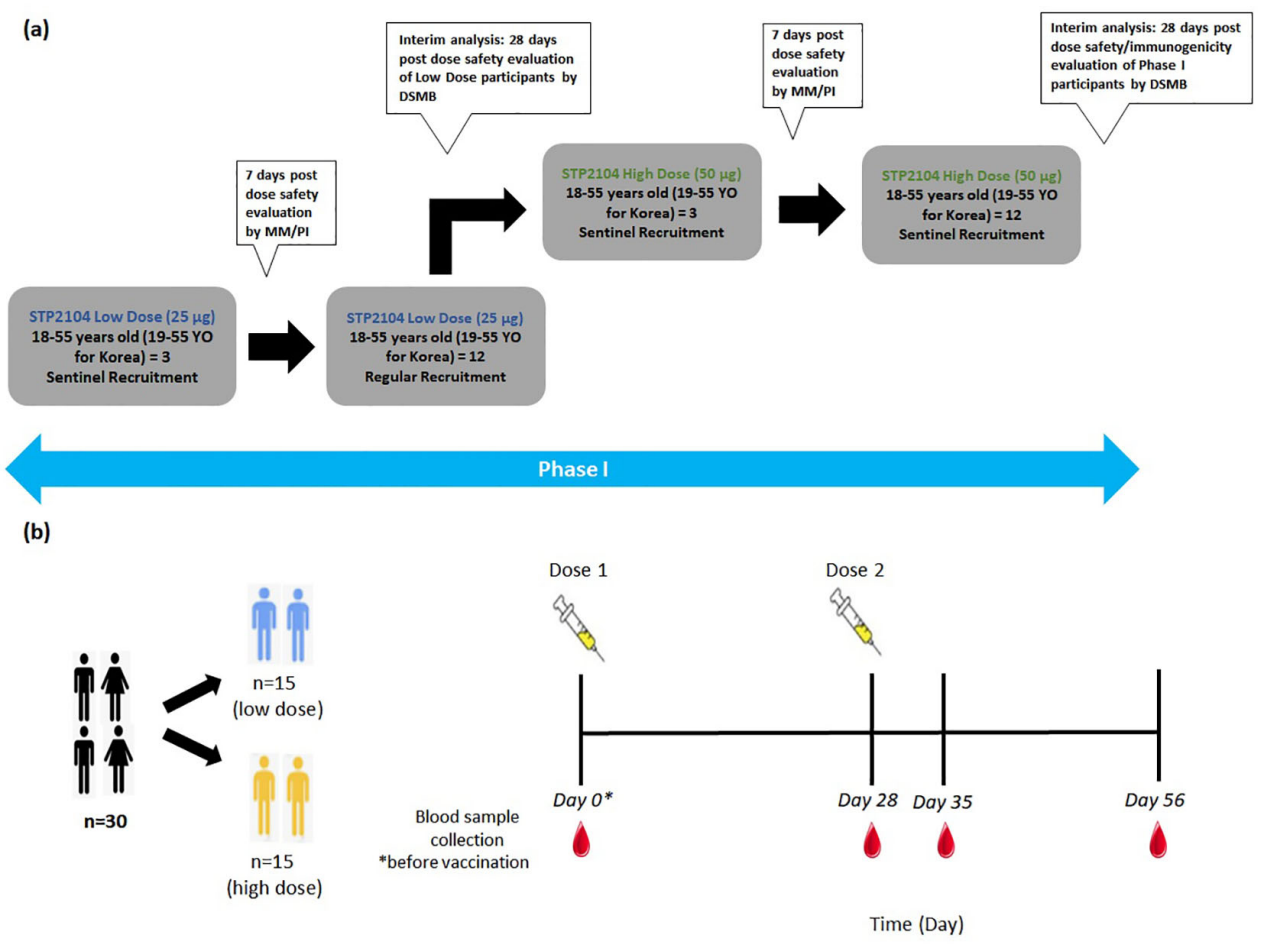
Schematic diagram depicting phase I clinical study design and dosing timepoints. **(a)** The study design schematic diagram depicts the outline of this multicenter, open-label, dose-escalation, phase I study to evaluate the safety and immunogenicity of a preventative COVID-19 vaccine STP2104 in healthy adults. After vaccination of the sentinel group with a low dose of 25 μg, followed by safety evaluation at 7 days post-dose, regular recruitment for low-dose vaccination was completed. Following interim analysis, vaccinations with a high dose of 50 μg were administered to the sentinel group, followed by a safety evaluation, regular recruitment vaccination, and interim analysis. **(b)** The dosing timepoint diagram depicts the different dose level cohorts and the timepoints of vaccination for both doses on day 0 and day 28, respectively. Blood samples were collected at the four timepoints on day 0, day 28 + 2, day 35 + 2, and day 56 + 2 and prior to vaccination if on the same day as the blood sample collection.

In this multicenter, open-label, dose-escalation, phase I study, 30 healthy adults (18 to 55 years of age and 19 to 55 years for subjects recruited in Korea) were enrolled as participants. Based on the order of enrollment, the first 15 participants were sequentially assigned to the low-dose-level cohort (25 μg), and the subsequent 15 participants were assigned to the high-dose-level cohort (50 μg) after dose escalation. Each dose-level cohort received two doses intramuscularly (same dose) separated by 28 days. Three participants in each dose-level cohort were assigned to the sentinel group with vaccinations administered at an interval of at least 24 h between each of the three subjects. If there were no adverse events (AEs) that met the temporary delaying criteria, vaccination proceeded for the next sentinel group subject until all individuals were vaccinated. A review was conducted of any AEs reported during the first 7 days after the first vaccination of the sentinel group, and if no AEs met the temporary delaying criteria, the remaining 12 participants in the dose-level cohort were enrolled. Between the low-dose cohort and the high-dose cohort, prior to dose escalation, an interim analysis of 7 days post-dose safety evaluation of the low-dose participants was conducted by the Data and Safety Monitoring Board (DSMB). If there were no safety issues, the study proceeded with dose escalation and vaccination of the high-dose-level cohort sentinel group. Blood collection timepoints were set at baseline (prior to the first vaccination), 4 weeks after the first vaccination, 1 week after the second vaccination, and 4 weeks after the second vaccination. The primary endpoints were to evaluate the safety and immunogenicity in all dose groups (**Figures 1a, b**).

### 2.7 IFN-γ enzyme-linked immunosorbent spot assay

The IFN-γ enzyme-linked immunosorbent spot (ELISpot) assay was performed on peripheral blood mononuclear cells (PBMCs) isolated from clinical participants and stored frozen using the Human IFN-γ ELISpot^pro^ kit (Mabtech, 3420-2AST-10, Nacka Strand, Sweden) according to the manufacturer’s protocol. Isolated PBMCs were stimulated with a synthesized SARS-CoV-2 (ancestral) peptide pool as a recall antigen. Each peptide was synthesized as a 15-mer with 9-mer overlapping with 1,273 full-length amino acids of the spike protein. All peptides were pooled as 5 according to the order from the N-term. Briefly, a 96-well ELISpot plate was washed with PBS, and each well was blocked with a cell culture medium for 30 min. The blocking medium was removed and 100 μL of diluted peptide pool, DMSO (negative control), and PHA (positive control; Sigma Aldrich, L1668, Burlington, MA, USA) was dispensed in the cell culture medium. Then, 100 μL of PBMCs (2 × 10^6^ cell/mL) rested in media for 18–20 h after thawing was added. After incubating the ELISpot plate in a CO_2_ incubator for 16 to 24 h, the cells were removed, each well was washed with PBS, and 100 μL of ALP-conjugated detection antibody was dispensed and incubated at room temperature for 2 h. After reaction, each well was washed with PBS, BCIP/NBT-plus Substrate (Mabtech, Nacka Strand, Sweden) was added, and the sample was reacted for 10 to 30 min before being washed with water to stop the reaction. After drying the plate at room temperature for 12 h, the number of spots was counted and analyzed using an ELISpot plate reader (Mabtech Astor™, Mabtech).

### 2.8 Plaque reduction neutralization titer 50

Neutralizing antibody (NAb) titration was conducted in the Korea National Institute of Infectious Diseases Good Clinical Laboratory Practice (KNIID GCLP-190). The method was adapted and implemented with modifications, based on a previously published paper (26). Briefly, Vero E6 (ATCC, catalog #CCL-81) was cultured in a 175T flask using DMEM (10% FBS, 1% Pen/Strep). All of the cell culture solution was removed from the 175T flask; then, 10 mL of DPBS was dispensed into the empty 175T flask, washed, and then removed. Following this, 3 mL of trypsin–EDTA was dispensed into a 175T flask and incubated for 3 min in a CO_2_ incubator. Then, 10 mL of 10% FBS DMEM was dispensed into the 175T flask after incubation, and we placed the cells separated from the bottom of the 175T flask into a 50-mL tube. After centrifugation at 1,500 rpm for 3 min, the supernatant was removed. The suspended cells were mixed in a 1:1 ratio using a 0.4% Trypan blue dye (Bio-Rad, 1450013, Hercules, CA, USA), and the cells were counted. Cells suspended at 2 × 10^5^/mL were dispensed, 1 mL each, into a 12-well plate. Serum inactivated at 65°C for 30 min was serially diluted two-fold from the stock solution using 2% FBS + 1% Pen/Strep + DMEM. The COVID-19 virus (BetaCoV/Korea/KCDC03/2020, NCCP #43326) diluted to 6 × 10^3^ PFU/mL was mixed with serum diluted by concentration in a 1:1 ratio. The virus was added to the diluted serum. The virus–serum mixture was incubated for 1 h in a CO_2_ incubator. After removing the medium from each well, 200 μL of the virus–serum mixture was inoculated into each well and incubated for 1 h in a CO_2_ incubator. After incubation, the mixed solution was removed, and 1 mL of overlay medium [1:1 mixture of 4% FBS MEM (2×) and 1.5% agar] was dispensed. This was incubated for 3 days in a CO_2_ incubator. The overlay medium was removed, and a crystal violet mixture [crystal violet solution, V5625; formaldehyde solution (252549 Sigma Aldrich); ethanol (Merck, 1.00983.1011, Rahway, NJ, USA)] (1 mL/well) was dispensed for at least 1 h at room temperature. After completely removing the agar medium and drying at room temperature, the number of plaques was measured. The NAb titer was defined as the dilution factor corresponding to 50% plaque reduction compared to the positive control (virus only). The average number of plaques was counted for each dilution. The 50% neutralizing dose (ND_50_) titer was calculated using Karber’s formula ND_50_ = log_10_ND_50_ (log_10_ND_50_ = m − Δ(∑p − 0.5; m: the highest dilution factor; Δ: log(dilution factor); ∑p: (number of plaque/average plaque no. of positive control) (27). The assay was set up based on comparison with the WHO International Standard (NIBSC code: 20/136, 4.00 IU/mL).

### 2.9 Statistical analysis

Statistical analyses were performed using one-way ANOVA with GraphPad Prism10 (Version 10.4.0, GraphPad Software, Inc., Boston, MA, USA).

## 3 Results

### 3.1 GLP repeated-dose toxicity studies showed that STP2104 was less toxic, with a NOAEL of 100 μg

The STP2104 vaccine was administered intramuscularly to SD rats weekly over 17 days at 25, 50, or 100 μg/animal to evaluate the systemic toxicity of the LNP-mRNA vaccine. The reversibility or progression of any treatment-related changes or delayed toxicity was assessed after 3 weeks of a treatment-free recovery period. Considering not only the use of lipid nanoparticle formulations of similar lipid composition in clinical trials but also the muscle mass/injection volume ratio in rats compared to humans, the risk for patients besides a local inflammatory reaction was deemed to be extremely low. Based on the no-observed-adverse-effect level (NOAEL) in GLP toxicity study, we set the human dosage in the clinical trial as two doses of 25 μg or 50 μg, respectively^2^.

### 3.2 STP2104 vaccination induced strong neutralizing antibody responses to the ancestral SARS-CoV-2 in humans

**Figure 2:**
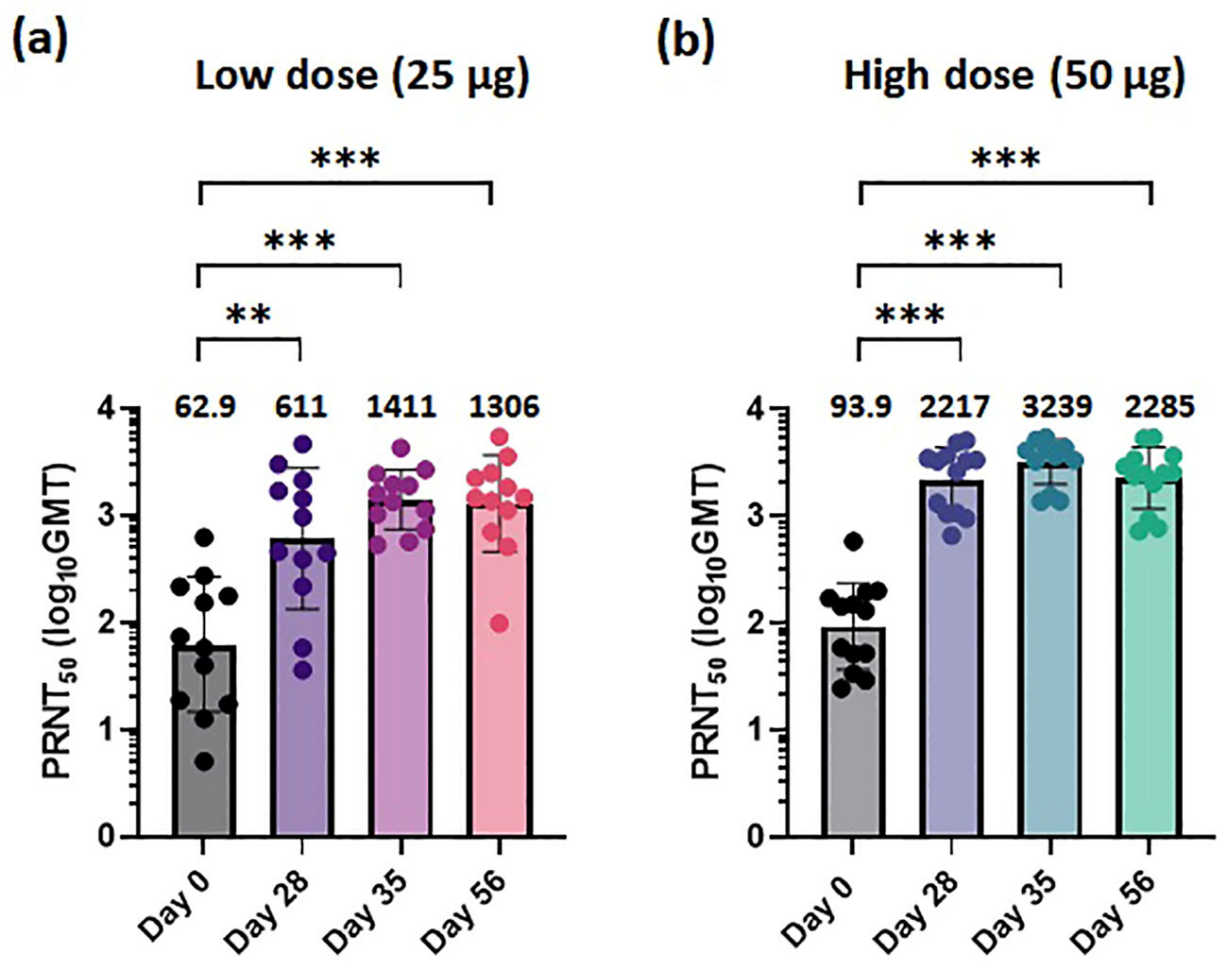
STP2104 vaccination induced a strong neutralizing antibody response to the ancestral SARS-CoV-2 in humans. The 30 clinical study participants received two doses of either 25 µg or 50 µg of STP2104 4 weeks apart (day 0 and day 28), with 15 individuals in each dose level cohort. For NAb analysis, serum samples from individuals were measured using the 50% plaque reduction neutralization test (PRNT_50_) for **(a)** low-dose (25 µg) and **(b)** high-dose (50 µg) cohorts. Sera were collected at the following timepoints: baseline, before the second immunization, and at 1 and 4 weeks after the second immunization. Data represent PRNT_50_ titers to ancestral SARS-CoV-2 with geometric mean and standard deviation. Statistical analysis was performed via one-way ANOVA using GraphPad Prism10 (Version 10.4.0, GraphPad Software, Inc., Boston, MA, USA) (**p < 0.01, ***p < 0.001).

We performed a NAb test using serum samples obtained from the 30 participants collected at four timepoints: baseline, 28 days after the first vaccination, 7 days after the second vaccination, and 28 days after the second vaccination. The plaque reduction neutralization test 50 (PRNT_50_) was conducted to measure NAb titers against the ancestral COVID-19 virus (BetaCoV/Korea/KCDC03/2020, NCCP #43326). After the first vaccination, the NAb titers significantly increased in both the 25-μg and 50-μg dose cohorts (**Figures 2a, b**). The titer was approximately three times higher in participants in the 50-μg cohort compared to the titer of participants in the 25-μg dose cohort. For both doses, the geometric mean (GMT) neutralizing titers increased at 28 days after the first vaccination compared to 7 days after the second vaccination and were measured at 611 and 2217, respectively. Four weeks post-second dose, STP2104 demonstrated NAb titers of 1,306 and 2,285 at the low dose and high dose, respectively, representing 20.8- and 24.3-fold increases from pre-dose.

From the *in-vivo* hACE2 transgenic (Tg) mouse challenge model, doses of 5 or 10 μg STP2104 showed higher protection efficacy. Similar to the result of *in-vivo* animal protection efficacy, a higher titer of PRNT_50_ is expected to be effective to prevent virus infection in humans. Our results show that sera from STP2104 vaccines can effectively neutralize the ancestral COVID-19 virus strain (S type) (**Figures 2a, b**).

### 3.3 STP2104 vaccination induced T-cell response to ancestral SARS-CoV-2 spike peptide stimulation in humans

**Figure 3:**
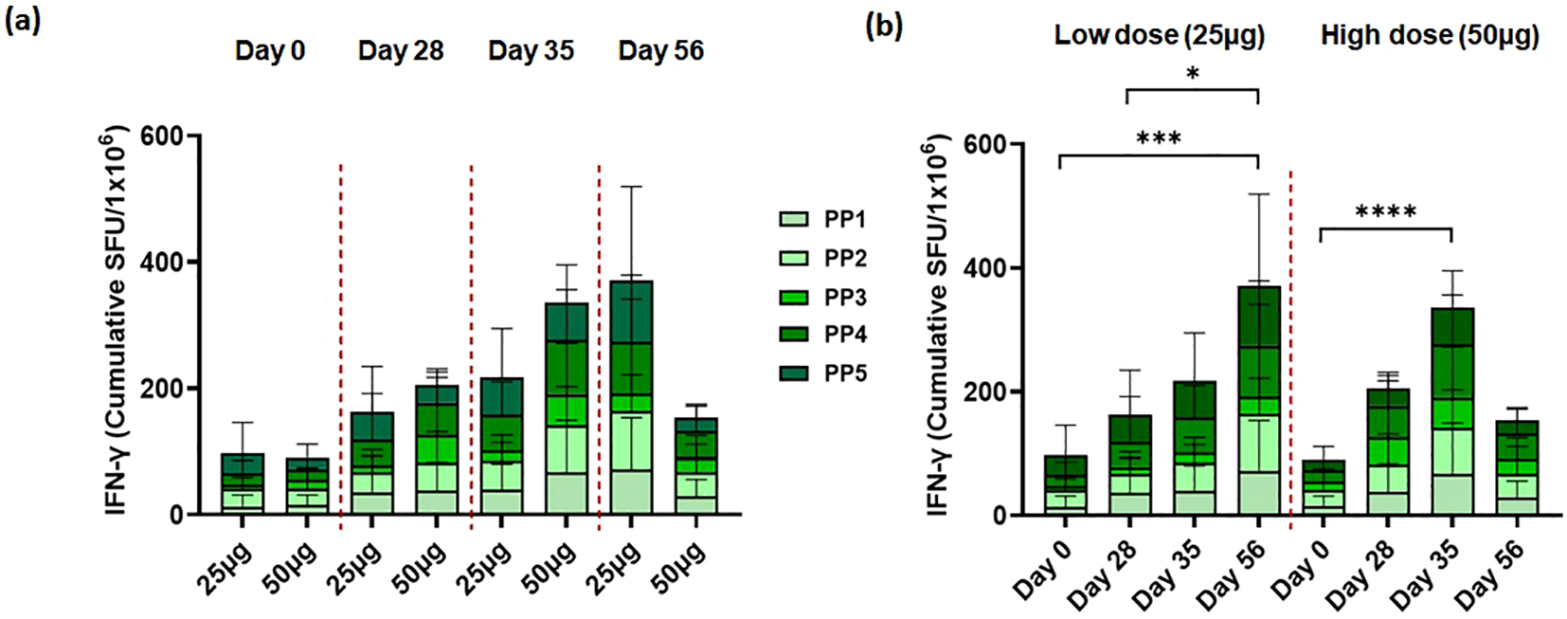
STP2104 vaccination induced T-cell response to ancestral SARS-CoV-2 spike peptide stimulation in humans. Thirty clinical participants received two doses of either 25 µg or 50 µg of STP2104 4 weeks apart (day 0 and day 28), with 15 individuals per dose level cohort, respectively. T-cell response was measured using IFN-γ ELISpot according to **(a)** frequency and time after dosing of low (25 µg) and high (50 µg) dose and **(b)** direct comparison between low- and high-dose cohorts. PBMCs were stimulated with ancestral-spike-originated 15-mer with 9-mer overlapping peptides. The IFN-γ secreting cells were quantified using an ELISpot assay and designated as spot-forming units (SFUs) in 2 × 10^5^ cells. Data represent the average and standard deviation of triplicates of each dose cohort. Statistical analysis was performed via one-way ANOVA using GraphPad Prism10 (Version 10.4.0, GraphPad Software, Inc., Boston, MA, USA) (*p < 0.05, ***p < 0.001, ****p < 0.0001).

We also analyzed T-cell immune responses to the ancestral SARS-CoV-2 spike. To confirm the T-cell immune response, PBMCs were collected on the same day as the NAb titer test. We stimulated PBMCs overnight with overlapping S peptide pools of peptides representing “peptide pool 1” (amino acids 1–249), “peptide pool 2” (amino acids 241–489), “peptide pool 3” (amino acids 481–729), “peptide pool 4” (amino acids 721–969), and “peptide pool 5’ (amino acids 961–1,273) of the SARS-CoV-2 spike protein. One month after the first vaccination, no significant spike-specific T-cell immune response was observed in either the 25-μg or 50-μg dose cohorts. After the second vaccination, spike-specific T-cell immune responses were observed in peptides 1, 3, and 4 in the 25-μg dose cohort and in peptides 2 and 4 in the 50-μg dose cohort. Compared to the 25-μg cohort, the 50-μg dose cohort showed a higher spike-specific T-cell immune response, and the 25-μg dose cohort showed a T-cell immune response that was maintained or increased until 28 days after booster (second) vaccination (**Figures 3a, b**). However, in the 50-μg dose cohort, the T-cell immune response was not sustained.

## 4 Discussion

We developed a CLS method using over 30 different novel cap analogs. The optimal novel 5′-cap analogue, SmartCap^®^ SC101, was selected and applied to the COVID-19 mRNA vaccine, STP2104. The SC101 is the proper 5′-cap analogue for synthesizing mRNA, leading to antigenic protein expression for prophylactic vaccine development. STP2104 showed high immunogenicity and protective efficacy in animal models. These results are consistent with cases where similar dose-dependent protective efficacy was confirmed with BNT162b2 (28) and mRNA-1273 (29). These studies evaluated the relationship between NAb titer and the protection efficacy of mRNA vaccines.

Furthermore, the STP2104 containing SC101-driven mRNA was evaluated for GLP toxicity, efficacy, and safety in a human clinical trial study. After confirming its safety through a GLP toxicology study, in phase I, STP2104 was administered intramuscularly in the deltoid muscle of COVID-19-naive participants; 15 participants received the low dose (25 μg) and 15 participants received the high dose (50 μg), with each cohort receiving two respective doses separated by 28 days (**Figure 1a**). The primary endpoints were to evaluate the safety and immunogenicity in all dose groups. During the reporting period of the trial, no SAEs were observed, and STP2104 demonstrated strong humoral and cellular immunities. In the clinical trial of the STP2104 mRNA vaccine, a candidate was designed against the SARS-CoV-2 ancestral strain. We evaluated the 25-µg and 50-µg dose cohorts showing that 28 days post-second dose, STP2104 demonstrated NAb titers of 1,306 and 2,285 at the low dose and high dose, respectively, representing 20.8- and 24.3-fold increases from pre-dose (day 0). This NAb titer is comparable to previously approved mRNA vaccines (30, 31). The normalized neutralization levels and immune protection efficacy show a strong non-linear relationship across different vaccine studies (32). The mRNA-1273 clinical trial study showed neutralization-titer-specific vaccine efficacy, which represents an increase in vaccine risk reduction. The higher NAb levels were correlated with a greater degree of protective efficacy (33). Therefore, the STP2104 prophylactic mRNA vaccine candidate, which induces potent NAb immune responses, is expected to offer strong protective efficacy and further reduce the severity of COVID-19.

In this study, we focused on evaluating the systemic immune response induced by the STP2104 mRNA vaccination in humans. A limitation of this study is that it did not assess the mucosal immune response, nor did it measure IgA antibody levels in human serum or another clinical specimen. Mucosal immunity remains a crucial aspect of protection against respiratory pathogens. Recent studies suggest that serum IgG and IgA levels do not accurately reflect mucosal immunity, as locally produced polymeric IgA plays a key role in neutralizing pathogens on mucosal surfaces (34). Several studies have highlighted the limitations of intramuscularly administered mRNA vaccines in eliciting robust mucosal immunity. While systemic immunity, characterized by strong IgG and T-cell responses, is effectively induced, mucosal immune responses, particularly secretory IgA (sIgA) production in the respiratory tract, remain suboptimal (35). BNT162b2 vaccination induced a transient increase in salivary IgA with neutralizing activity, but this response diminished over time, indicating limited and short-lived mucosal protection (35, 36). Similarly, intramuscular mRNA vaccination led to a modest increase in RBD-specific IgA1 in children, whereas natural infection resulted in higher levels of IgA2, which is more abundant in mucosal tissues. These findings suggest that while intramuscular mRNA vaccines can elicit some degree of mucosal immunity, they may not be sufficient to provide long-lasting protection in the respiratory tract (37). In addition, IgA responses in saliva wane within 6 months post-infection or vaccination, suggesting that current vaccines do not provide sustained mucosal immunity (38).

Furthermore, evidence supports the necessity of alternative vaccine strategies to enhance mucosal immunity. Previous research has shown that while mRNA vaccines induce strong systemic responses, they generate minimal mucosal immunity, as evidenced by low SARS-CoV-2-specific IgA and T-cell responses in bronchoalveolar lavage fluid. Additionally, animal studies have demonstrated that combining systemic mRNA vaccination with an intranasal adenoviral vector booster significantly enhances mucosal immune responses and neutralizing activity against Omicron variants (39). This suggests that mucosal booster vaccines could be an effective approach to strengthening protection against breakthrough infections.

However, several variants of SARS-CoV-2 continue to emerge, with some capable of evading the host’s immune response. Therefore, efficient vaccine development strategies should focus on inducing cross-protective immune responses against SARS-CoV-2 variants and, potentially, even universal pan-sarbecoviruses. Although spike S1-specific homologous NAbs were induced, they lacked cross-neutralizing activity. In contrast, NAb and functional binding Abs against the chimera vaccine including the receptor binding domain (RBD), N-terminal domain (NTD), and S2 elicited fully protective effects (40), and non-NAb activity targeting spike S2-FcγR4 was found to be cross-protective in wild-type mice (41). This is supported by another study demonstrating that FcγRs bound to vaccine-induced antibodies and that alveolar macrophages actively contributed to protection against SARS-CoV-2 variants in a mouse model (42).

To overcome these limitations and provide broader protection against SARS-CoV-2 and its evolving variants, strategies such as intranasal vaccine platforms or mucosal booster approaches may be required. Given that current COVID-19 vaccines do not fully prevent infection and transmission, there is growing emphasis on next-generation vaccines that elicit strong mucosal immunity. Therefore, future research and development should prioritize optimizing immunogens, immunization routes to activate mucosal responses, and vaccination regimens that enhance protection against respiratory viral infections.

In our results, there were no significant differences in the T-cell response to ancestral SARS-CoV-2 spike peptide stimulation in humans between the low-dose and high-dose groups. Consistent with our findings, previous studies have shown that high antigen doses can attenuate vaccine-specific T-cell responses. For instance, the administration of a high dose of HIV antigen, in combination with cationic liposomal adjuvants, resulted in a reduction in polyfunctional T cells producing IFN-γ and TNF-α compared to lower doses. In a mouse model of tuberculosis, high antigen doses negatively affected the efficacy of post-exposure vaccines against tuberculosis infection, a phenomenon attributed to terminal differentiation and the decreased functional avidity of T cells (43). Initial T-cell activation is antigen-quantity-dependent, but beyond a certain threshold, the response becomes saturated. According to studies, the activation of both CD8^+^ and CD4^+^ T cells increases with increasing antigen concentration, but beyond a certain critical point, the response no longer increases. The amount of antigen determines the T-cell response; however, beyond a certain level, adding more antigen does not further enhance the response. This suggests that when antigen affinity exceeds a certain threshold, T-cell responses become saturated, and further increases in antigen concentration may not strengthen the response (44). In addition, PD-1 and TIGIT are immune checkpoint receptors that are expressed on CD8^+^ T cells repeatedly exposed to antigens, leading to a decline in cell functionality. This suppresses the immune response and prevents T cells from effectively controlling the virus. Increased levels of PD-1 and TIGIT, markers of exhaustion and aging, in CD8^+^ T cells after vaccine administration suggest that vaccine responses in HIV-1-infected individuals are associated with changes related to immune cell fatigue and aging (45). Therefore, CD8^+^ T cells with increased PD-1 and TIGIT are likely in a state of exhaustion and aging, which could be a major factor in their inability to effectively suppress viral replication. Given that high antigen doses can induce T-cell exhaustion and aging, the precise modulation of vaccine dosing is essential to optimize T-cell responses, as excessive antigen doses can lead to the clonal deletion, immune tolerance, terminal differentiation, or exhaustion of T cells (46).

In conclusion, the safety, NAb titer, and cellular immunity interim results support the potency and safety of the SmartCap^®^ SC101 and the effectiveness of STP2104 in inducing NAb titers against SARS-CoV-2. This multicenter, open-label, dose-escalation, phase I study with 30 healthy adults demonstrates the first proof of SC101 safety and efficacy in humans. In addition, this report emphasizes the importance of optimal capping selection. Furthermore, the full story of reactogenesis, safety, and tolerability based on the interim clinical study report (CSR) of this clinical study will be presented subsequently.

## Data Availability

The raw data supporting the conclusions of this article will be made available by the authors, without undue reservation.

## Footnotes

1 STP2104 Vaccine: 17-day Toxicity Study by the Intramuscular Route in SD Rats Followed by a 3-week Recovery Period. 2022, AnaPath Research: Santa Perpètua de Mogoda, Barcelona, Spain. Unpublished report.

2 STP2104 Vaccine: 17-day Toxicity Study by the Intramuscular Route in SD Rats Followed by a 3-week Recovery Period. 2022, AnaPath Research: Santa Perpètua de Mogoda, Barcelona, Spain. Unpublished report.
